# SDF-1/CXCR7 Chemokine Signaling is Induced in the Peri-Infarct Regions in Patients with Ischemic Stroke

**DOI:** 10.14336/AD.2017.1112

**Published:** 2018-04-01

**Authors:** Yu Zhang, Hongxia Zhang, Siyang Lin, Xudong Chen, Yu Yao, XiaoOu Mao, Bei Shao, Qichuan Zhuge, Kunlin Jin

**Affiliations:** ^1^Department of Neurosurgery, the First Affiliated Hospital, Wenzhou Medical University, Wenzhou, Zhejiang 325000, China.; ^2^Department of Pharmacology and Neuroscience, University of North Texas Health Science Center at Fort Worth, Texas 76107, USA.; ^3^Zhejiang Provincial Key Laboratory of Aging and Neurological Disorder Research, the First Affiliated Hospital, Wenzhou Medical University, Wenzhou, Zhejiang 325000, China.; ^4^Department of Neurosurgery, Huashan Hospital, Fudan University, Shanghai 200040, China.; ^5^Buck Institute for Age Research, Novato, California 94945, USA

**Keywords:** SDF-1, CXCL12, CXCR4, CXCR7, chemokine, penumbra, ischemia, stroke, patient, human

## Abstract

Stromal-derived factor-1 (SDF-1, also known as CXCL12) and its receptors CXCR4 and CXCR7 play important roles in brain repair after ischemic stroke, as SDF-1/ CXCR4/CXCR7 chemokine signaling is critical for recruiting stem cells to sites of ischemic injury. Upregulation of SDF-1/CXCR4/CXCR7 chemokine signaling in the ischemic regions has been well-documented in the animal models of ischemic stroke, but not in human ischemic brain. Here, we found that protein expression of SDF-1 and CXCR7, but not CXCR4, were significantly increased in the cortical peri-infarct regions (penumbra) after ischemic stroke in human, compared with adjacent normal tissues and control subjects. Double-label fluorescence immunohistochemistry shows that SDF-1 and CXCR4 proteins were expressed in neuronal cells and astrocytes in the normal brain tissue and peri-infarct regions. CXCR7 protein was also observed in neuronal cells and astrocytes in the normal cortical regions, but predominantly in astrocytes in the penumbra of ischemic brain. Our data suggest that ischemic stroke in human leads to an increase in the expression of SDF-1 and CXCR7, but not CXCR4, in the peri-infarct cerebral cortex. Our findings suggest that chemokine SFD-1 is expressed not only in animal models of stroke, but also in the human brain after an ischemic injury. In addition, unlike animals, CXCR7 may be the primary receptor of SDF-1 in human stroke brain.

Stromal cell-derived factor 1 (SDF-1), also known as C-X-C motif chemokine 12 (CXCL12), is a chemokine protein that ubiquitously expressed in many tissues and all cell types in the central nervous system (CNS)[1]. SDF-1 signaling is initiated by ligation of the chemokine with its G-protein-coupled receptor C-X-C chemokine receptor type 4 (CXCR-4; also known as fusin or CD184) [2] that is expressed in neurons, microglia, astrocytes, bone marrow stromal cells (BMSCs), and some other stem cells [3]. Recently, studies revealed that SDF-1 also binds and signal through the CXCR7 receptor (also known as RDC-1 or ACKR3) [4], which exhibits a distinctly higher binding affinity for SDF-1 than CXCR4 [4].

SDF-1/CXCR4/CXCR7 signaling axis is involved in many biological processes, including cardiovascular organogenesis, hematopoiesis, immune response, tumor growth and metastasis, as SDF-1 is a major factor of stem cell homing [1, 5-7], a process where the cells in the circulation migrate to the target tissues [8]. SDF-1 and CXCR4 are constitutively expressed in the brain. However, after focal ischemic stroke, SDF-1 is upregulated in the ischemic regions, especially in the per-infarcted regions (penumbra) [9]. The level of SDF-1 expression peaks at 3-7 days and remains elevated until 6 weeks [9]. In addition, CXCR7 expression increases rapidly in the penumbra [10]. Increased SDF-1 has the ability of recruiting CXCR4^+^ stem/progenitor cells to the ischemic tissue, which include hematopoietic stem cells (HSPCs) [11, 12], bone marrow mesenchymal stem cells (BMSCs) [9, 13], endothelial progenitor cells (EPCs)[14, 15], and neural stem/progenitor cells (NSCs) [16, 17]. Stem cell homing is critical for functional recovery after ischemic stroke in animals. Consistently, a recent study shows that SDF-1 level is also increased in the serum of patients with stroke and the serum SDF-1 change is positively correlated with infarct volume and severity of stroke in patients [18-20]. Taken together, these findings suggest that SDF-1/CXCR4/CXCR7 signaling axis plays an important role in neurogenesis and angiogenesis after ischemic stroke. Therefore, a better understanding of how SDF-1 is expressed and functions would be beneficial in developing specific therapeutic strategies for ischemic stroke. However, these findings are mainly based on rodent brains. Little is known about how SDF-1/CXCR4/CXCR7 signaling axis responds to acute ischemic stroke in the human brain.

In this study, we found that expression of SDF-1 and CXCR7, but not CXCR4, were significantly increased in the cortical peri-infarct regions after ischemic stroke, compared with adjacent normal tissues and control subjects. Double immunolabeling shows that SDF-1 and CXCR7 were expressed in neuronal cells and astrocytes in the normal tissues. However, unlike SDF-1, CXCR7 was predominantly located in the reactive astrocytes in the peri-infarct region in brain specimens of stroke patients. Our data suggest that SDF-1 play a role in brain repair after ischemic stroke in human through a CXCR7 dependent mechanism.

## MATERIALS AND METHODS

### Human Brain Tissue

Human stroke specimens (*n* = 9) were obtained at biopsy performed for diagnostic purposes, with informed consent and in accordance with protocols approved by the Institutional Research Review Board at Huashan Hospital of Fudan University, China. Cerebral cortical infarcts were confirmed by histopathology in six of nine patients, who were included in the present study; the remaining three of nine biopsied patients were excluded because of other diagnoses (one each with cerebellar infarct, intracerebral hemorrhage, and intraventricular hemorrhage). More details, such as age, sex, duration of symptoms and infarct sites, have been documented in our previous study [21]. Four normal human brain specimens without clinical or postmortem evidence of neurological diseases were obtained from the Brain and Tissue Bank for Developmental Disorders of the National Institute of Child and Health and Human Development (University of Maryland, Baltimore, MD, USA).

### Immunohistochemistry

Human brain specimens were embedded in paraffin and cut in 6-µm sections, which were deparaffinized with xylene and rehydrated with ethanol, following antigen retrieval with antigen unmasking solution (Vector Laboratories, Burlingame, CA, USA) according to the manufacturer’s instructions. Endogenous peroxidase activity was blocked by incubation in 1% H_2_O_2_ at room temperature for 30 min. After several washes with PBS, sections were incubated in blocking solution (2% goat serum, 0.3% Triton X-100 and 0.1% bovine serum albumin in PBS) for 1 hr at room temperature. Primary antibody rabbit polyclonal anti-SDF-1α (Novus Biologicals, USA; 1:200), rabbit anti-CXCR7 (Proteintech Group, IL, USA; 1:500) or rabbit anti- Anti-CXCR4 (Millipore, Billerica, MA, USA; 1:200) was added in blocking buffer and incubated with sections at 4 °C overnight. Sections were then washed with PBS and incubated with biotinylated goat anti-rabbit (Santa Cruz Biotechnology, Santa Cruz, CA, USA; 1:200) for 1 hr at room temperature. Avidin-biotin complex (Vector Elite; Vector Laboratories) and a diaminobenzidine or nickel solution (Vector Laboratories, USA) were used to obtain a visible reaction product. The slide examiners were blinded to the source of the specimen (stroke *vs*. control). A Nikon microscope and a Nikon digital color camera were used for examination and photography of the slides, respectively.

### Double immunostaining

Double immunostaining was performed on brain sections as previously described [22, 23]. The primary antibodies used, in addition to antibodies describe above, included mouse monoclonal anti-GFAP and mouse anti-NeuN (Millipore, Billerica, MA, USA; 1:200). The secondary antibodies were Alexa Fluor 488-, 594-, or 647-conjugated donkey anti-mouse or anti-rabbit IgG (Molecular Probes, 1:200-500). Slides were mounted using proLong Gold antifade reagent with DAPI (Molecular Probes). Fluorescence signals were detected using an LSM 510 NLO Confocal Scanning System mounted on an Axiovert 200 inverted microscope equipped with a two-photon Chameleon laser. Selected images were viewed at high magnification, and 3-dimensional images were constructed using IMARIS software. Controls included omitting either the primary or secondary antibody.

**Figure 1.**
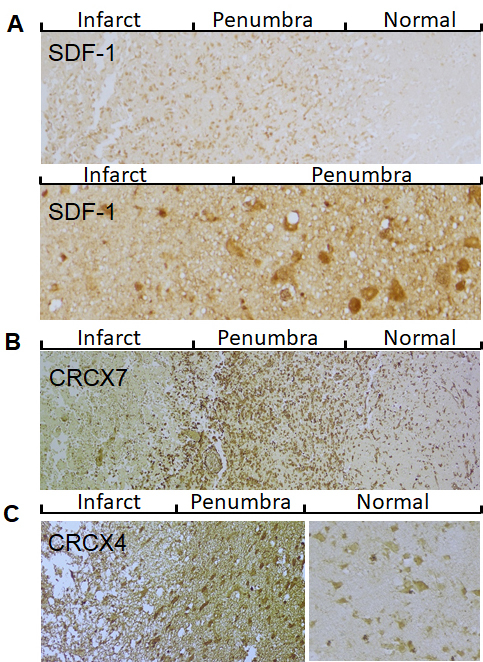
Expression pattern of SDF-1/CXCR4/CXCR7 in post-stroke human brain. A) Representative images show that SDF-1 expression in cerebral cortex of infarcted brain. Top panel: low magnification; Bottom panel: high magnification. B) CXCR7 immunocytochemistry in the peri-infarct region (penumbra) and adjacent normal tissue. C) CXCR4 immunocytochemistry in the cortical penumbra and adjacent normal tissue.

**Figure 2.**
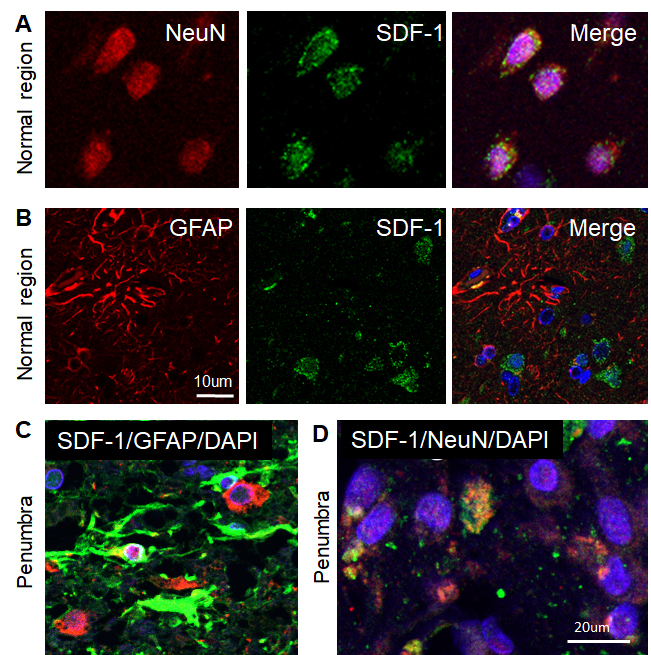
Phenotypes of SDF-1-positive cells in the human ischemic brain. A-B) Confocal image of representative immunofluorescent staining for NeuN (A) or GFAP (B) (Alexa Fluor 594, red), SDF-1 (Alexa Fluor 488, green), nuclei (DAPI, blue), and merged image from adjacent normal regions of human ischemic stroke brain. C) Merged confocal images of double-label immunohistochemistry in the peri-infarct region (penumbra) of the human ischemic brain section using anti-GFAP (green) and anti-SDF-1 (red). D) Merged confocal images of double-label immunohistochemistry in the penumbra on the human ischemic brain using anti-NeuN (red) and anti-SDF-1 (green). DAPI (blue) was used for nuclei counterstains.

## RESULTS

To determine the expression pattern of SDF-1 protein, immunocytochemistry was performed on the ischemic brain sections from patients with ischemic stroke using anti-SDF-1 antibody. As shown in Fig. 1A, SDF-1-immunopositive cells were predominantly localized in the cortical peri-infarct region (penumbra) and infarct area, but a little in the adjacent normal region in brain specimens of stroke patients, suggesting that SDF-1 protein is induced in the ischemic regions after ischemic stroke. Next, we examined the expression patterns of second SDF-1 receptor CXCR7, Like SDF-1, CXCR7 protein expression was significantly increased in the penumbra of human ischemic brain, compared with the adjacent normal regions. However, the expression of CXCR7 was low in the infarct core (Fig. 1B). Interestingly, the expression of CXCR4 protein in the cortical penumbra was not significantly increased, compared with the adjacent normal region, and essentially absent from the infarct core (Fig. 1C). These findings suggest that CXCR7, rather than CXCR4, is the primary receptor for SDF1 in the ischemic brain in human.

**Figure 3.**
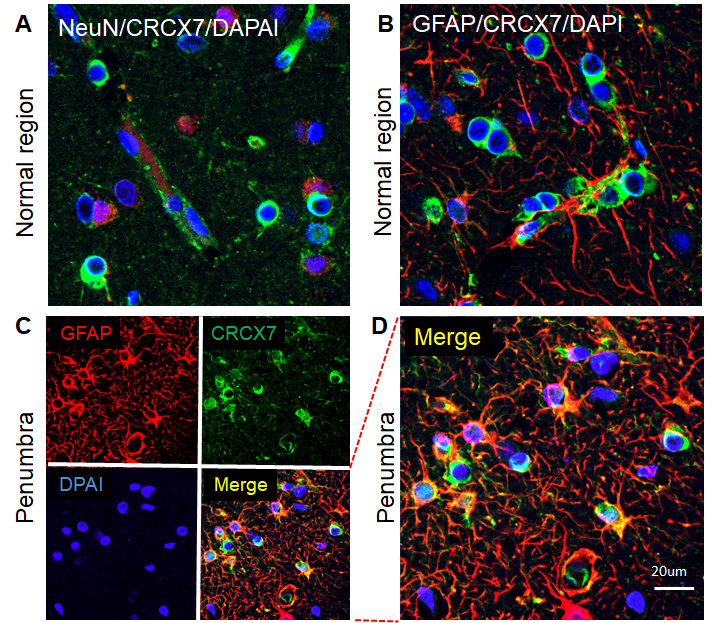
Phenotypes of CXCR7-positive cells in the human ischemic brain. A) Merged confocal image of double-label immunohistochemistry on the normal region of the human ischemic brain section using anti-NeuN (red) and anti- CXCR7 (green). B) Merged confocal image of double-label immunohistochemistry on the normal region of the human ischemic brain section using anti-GFAP (red) and anti- CXCR7 (green). C) Double immunocytochemistry was performed on the ischemic brain sections in the penumbra using anti-GFAP (red) and anti-CXCR7 (green). The images were recorded using a 2-photon confocal microscope. D) Higher magnification view of merged confocal image in panel C. DAPI (blue) was used for nuclei counterstains.

**Figure 4.**
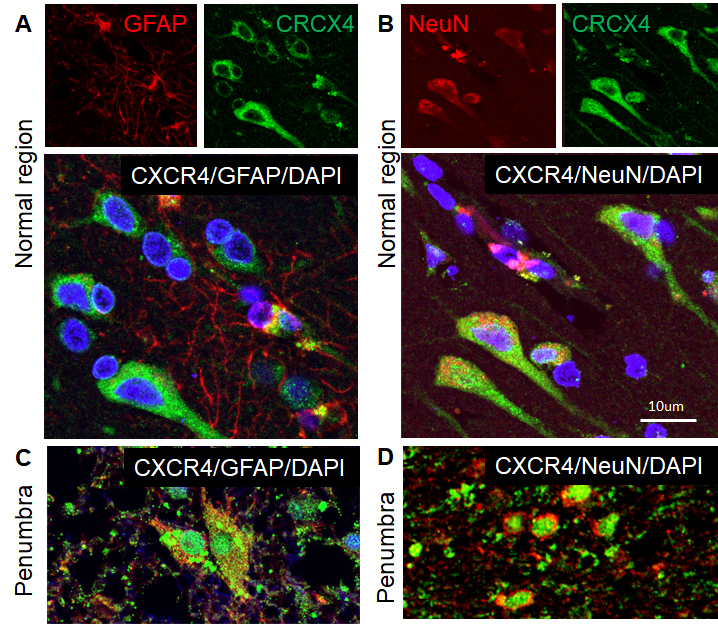
Phenotypes of CXCR4-expressed cells in the human ischemic brain. Double immunocytochemistry was performed on the ischemic brain sections and the images were recorded using a 2-photon confocal microscope. Representative images show that CXCR4 (green) was expressed in GFAP-positive astrocytes (red) in the normal region (A) and penumbra (C), and NeuN-positive neuronal cells (red) in the normal region (B) and penumbra (D) of human ischemic brain. DAPI (blue) was used for nuclei counterstains.

To examine the phenotypes of SDF-1-positive cells in the brain of human ischemic stroke, double-label fluorescence immunohistochemistry was performed. As shown in Fig. 2, SDF-1 was primarily localized to neurons and astrocyte either in brains of patients with stroke or normal brains, which were identified by staining for neuronal nuclei (NeuN) and GFAP-expressing astrocytes. Although CXCR7 protein was expressed in neuronal cells and astrocytes in the normal brain and adjacent normal tissue in brain specimens of stroke patients (Fig. 3A and B), CXCR7 expression was mainly in the astrocytes in the penumbra of human stroke brain (Fig. 3C and D). While CXCR4 was expressed in neuronal cells and astrocytes in the normal tissue and per-infarcted regions (Fig. 4). The expression of SDF-1, CXCR7 and CXCR4 in neuron and astrocyte, these proteins were also expressed in the vascular cells in the brain of patients with ischemic stroke (data not shown).

## DISCUSSION

The major finding of this study is that ischemic stroke in human leads to an increase in the expression of SDF-1 and CXCR7, but not CXCR4, in the peri-infarct cerebral cortex compared with the adjacent normal brain and ischemic core.

Stem cells are a source of paracrine and structural regeneration for brain damaged by acute ischemic stroke. Therefore, proper homing of stem cells, including HSCs, BMSCs, EPCs and NSCs, to ischemic brain, is critical for the restoration of the injured neural tissues and improved functional outcomes. Although the mechanism underlying stem cell homing to ischemic regions is unclear, the overexpression and secretion of SDF-1 by ischemic tissues could develop a gradient in circulation and guides CXCR4-postive stem cell recruitment from bone marrow and neurogenic niches in the brain to the injured site [24]. Indeed, study has shown that deficiency of CXCR-4 significantly decrease the migration of BMSCs toward the ischemic region, indicating the SDF-1/CXCR-4 play an important role in regulating the homing of BMSCs [9, 18, 25-28]. In addition, SDF-1/CXCR4 increased the radial migration of NSCs from the subventricular zone (SVZ) toward the infarct areas in a dose-dependent manner [29]. Blockade of the CXCR4 or CXCR7 could disrupt the migration of NSCs, leading to a failure of the newborn neurons to migrate to the ischemic tissue in animal models of stroke [30]. In human, higher increase in CXCR4-positive stem cell number and lower increase in CXCR4-positive cells in peripheral blood in acute stroke patients correlated with initially worse neurological status. However, increased CXCR4-positive cell induction in peripheral blood of acute stroke patients correlated with better functional/neurological status after the 6-month follow-up [31]. Similar to the findings in animals, our data show that SDF-1 is increased in the penumbra of patients with ischemic stroke, indicating that the function of up-regulated SDF-1 could recruit different types of stem cells at different locations to ischemic regions. Majority of cells that expressed SDF-1 are neurons, astrocyte and endothelial cells, suggest that SDF-1 may be released from these cells after ischemic stroke, but primarily secreted by astrocytes and endothelial cells in animal models of ischemic stroke [9].

Interestingly, our data also showed that CXCR7, but not CXCR4, was significantly increased in the peri-infarcted regions in human stroke brain. Specifically, we found that these CXCR7-positive cells in the peri-infarcted regions are predominantly astrocytes in human. Consistently, previous studies have documented that, astrocytes show the rare expression of CXCR4 in the developing and injured brain, whereas throughout the brain a much larger subset of astrocytes seem to express CXCR7 [10, 32, 33]. The origin of the CXCR7-psotive astrocytes in the cortical penumbra is unknown. It is possible that these CXCR7-positive mature cells are derived from stem cells migrated from other regions. However, CXCR7-positive astrocytes most likely are resident cells. Therefore, it is consequently feasible to assume that the predominant role of CXCR7 in astrocytic SDF-1 signaling could be different from stem cell homing. Supported evidence includes that RNAi-mediated depletion of CXCR7 silenced SDF-1 signaling in astrocytes [34], and that SDF-1 *in vitro* acts as a growth factor for astrocytes by stimulating their proliferation [35], a phenomenon that could represent the basis of pathological conditions such as gliosis or reactive astrogliosis [36]. Peri-infarct astrocytes undergo reactive astrogliosis after ischemic stroke, which plays key roles in modulating the adaptive responses in neurons. Our data suggest that SDF-1/CXCR7 signaling is likely to be related to glial proliferation in the peri-infarcted regions after ischemic stroke. Further study should be performed to determine whether SDF-1/CXCR7 signaling is truly involved in astrogliosis or glial scar formation in patients with ischemic stroke.
